# Autoimmune Pemphigus: Latest Advances and Emerging Therapies

**DOI:** 10.3389/fmolb.2021.808536

**Published:** 2022-02-04

**Authors:** Yen Loo Lim, Gerome Bohelay, Sho Hanakawa, Philippe Musette, Baptiste Janela

**Affiliations:** ^1^ Department of Dermatology, National Skin Centre, Singapore; ^2^ Department of Dermatology and INSERM U1125, Avicenne Hospital, Bobigny, France; ^3^ A*STAR Skin Research Labs (ASRL), Agency for Science, Technology and Research (A*STAR), Singapore; ^4^ Skin Research Institute of Singapore (SRIS), Agency for Science, Technology and Research (A*STAR), Singapore; ^5^ A*STAR Infectious Diseases Labs, Agency for Science, Technology and Research (A*STAR), Singapore; ^6^ Singapore Immunology network, Agency for Science, Technology and Research (A*STAR), Singapore

**Keywords:** pemphigus, pemphigus treatment, autoimmunity, autoimmune bullous diseases, advance in pemphigus

## Abstract

Pemphigus represents a group of rare and severe autoimmune intra-epidermal blistering diseases affecting the skin and mucous membranes. These painful and debilitating diseases are driven by the production of autoantibodies that are mainly directed against the desmosomal adhesion proteins, desmoglein 3 (Dsg3) and desmoglein 1 (Dsg1). The search to define underlying triggers for anti-Dsg-antibody production has revealed genetic, environmental, and possible vaccine-driven factors, but our knowledge of the processes underlying disease initiation and pathology remains incomplete. Recent studies point to an important role of T cells in supporting auto-antibody production; yet the involvement of the myeloid compartment remains unexplored. Clinical management of pemphigus is beginning to move away from broad-spectrum immunosuppression and towards B-cell-targeted therapies, which reduce many patients’ symptoms but can have significant side effects. Here, we review the latest developments in our understanding of the predisposing factors/conditions of pemphigus, the underlying pathogenic mechanisms, and new and emerging therapies to treat these devastating diseases.

## Introduction

Autoimmune bullous diseases (AIBDs) occur when self-reactive immune responses target the basement membrane or desmosomes of the skin and mucous membrane, leading to loss of epithelium integrity and varying severities of painful blistering (Schmidt et al., 2019). As a primary role of the skin is to protect from invading microorganisms, patients with bullous diseases can suffer recurrent, and potentially life-threatening infections, as well as chronic pain and lowered quality of life (Hsu et al., 2016). AIBDs are separated in two main subtypes, pemphigus and pemphigoid diseases. Pemphigus, from the Greek word “pemphix” (blister), is a heterogenous group of AIBDs affecting the stratified squamous epithelia, responsible for intraepithelial blistering, which is caused by inter-keratinocytic deposits of autoantibodies. Pemphigoid group in contrast affects the subepithelial layers of the skin and/or mucous membranes, responsible for subepithelial blistering, and thus exhibit distinct clinical features. In comparison with subepithelial AIBDs, most types of pemphigus do not involve the basement membrane. Among the pemphigus family are those more common variants, pemphigus vulgaris (PV) and pemphigus foliaceus (PF), as well as the rarer paraneoplastic pemphigus (PNP), pemphigus herpetiformis (PH), and IgA pemphigus (Joly and Litrowski, 2011; Kasperkiewicz et al., 2017).

Although clinically and aetiologically distinct, all pemphigus variants induce the formation of flaccid blisters and erosions on the skin and/or mucous membranes, and are characterised histologically by acantholysis, in which keratinocytes separate from each other (Amagai et al., 1991). In this case, acantholysis is caused by autoimmune disruption of the desmosomes - specialized adhesive protein complexes that connect neighbouring keratinocytes to each other - as a result of the generation and deposition of IgG auto antibodies against the desmosomal structural proteins desmoglein (Dsg) 1 and/or Dsg3 (Amagai et al., 1991). While the main antigenic targets of these autoimmune skin conditions were discovered several decades ago, our understanding of the factors underpinning the initiation and progression of pemphigus remains incomplete. Moreover, despite recent advances in the treatment of pemphigus, many patients continue to suffer repeated relapses of their condition and/or treatment side-effects, and a significant proportion of cases remain refractory to currently available therapies. Pemphigus mortality rates is elevated compared to the general population (Kridin et al., 2017; Jelti et al., 2019).

In this review we will bring together recent progress in our understanding of the aetiology and pathogenic mechanisms of pemphigus and note those areas in which our knowledge remains incomplete. We will also discuss the latest advances and clinical trials aiming to offer better treatment of this challenging set of diseases and highlight key areas for future research and development.

## The Pemphigus Family of Autoimmune Skin Diseases: Clinical Features and Diagnosis

All patients with pemphigus variants exhibit autoantibodies against desmosomal structural proteins, but the discrimination of each variant is based on specificities in clinical presentation and/or histological features or in most commonly auto-antibodies encountered (Hertl et al., 2015) (Table 1). Understanding the features that distinguish between variants and sub-variants is necessary to allow accurate and rapid diagnosis; moreover, it is hoped that, as we increased our understanding, variant-specific therapies will begin to emerge as part of personalised treatment strategies that take into account any comorbidities in this fragile patient population.

**TABLE 1:** Types of pemphigus.

Clinical variant	General frequency	Ig subtype	Autoantigen	Clinical hallmarks
Pemphigus vulgaris	70 to 90%	IgG	Dsg3, Dsg1	Mainly oral mucosal lesions affecting the gingiva, palate, floor of the mouth, buccal mucosa, lips, tongue, nasal, pharyngeal, laryngo-oesophageal, urethral, genital (glans penis, vulva), perianal and conjunctival mucosae
Pemphigus foliaceus	10 to 30%	IgG	Dsg1	Flaccid and fragile blisters involving skin; no mucous lesions; erythroderma, puff pastry-like scales and crusts affecting seborrheic areas (scalp, face, presternal, interscapular skin)
Paraneoplastic pemphigus	5%	IgG	Dsg3, Dsg1, desmoplakin I and II, envoplakin, periplakin, plectin, BP230, BP180, A2ML1, Dsc1, epiplakin	Severe mucocutaneous lesions with erosions and ulcers involving tongue, lips and vermillion border, nasal, conjunctival and genital mucosa; skin involvement are polymorphic, with pustular, erythema multiform-like or lichenoid lesions; association with lymphoproliferative disorder, lymphoma, leukaemia, carcinomas, sarcomas
Pemphigus vegetans	1–2%	IgG	Dsg3, Dsg1, periplakin	Hypertrophic papillomatous vegetative skin lesions affecting intertriginous sites (axillae, inframammary folds, umbilicus, inguinal creases, anogenital skin, scalp, neck), flaccid blisters and erosions with fetid-odoured
IgA pemphigus	<2%	IgA	Dsg3, Dsg1, Dsc1	Annular erosive plaques, pustules affecting the skin of the trunk, proximal extremities, and intertriginous regions; uncommon mucosal involvement
Pemphigus herpetiformis	<2%	IgG	Dsg3, Dsg1, Dsc	Pruritic herpetiform blisters, annular urticarial plaques with or without erosions, scaly erosive patches mimicking eczema

Dsg, desmoglein; Dsc, desmocollin; BP230 or 180, bullous pemphigoid 230 or 180; A2ML1, α-2 microglobulin-like 1.

### Pemphigus Vulgaris

**FIGURE 1:**
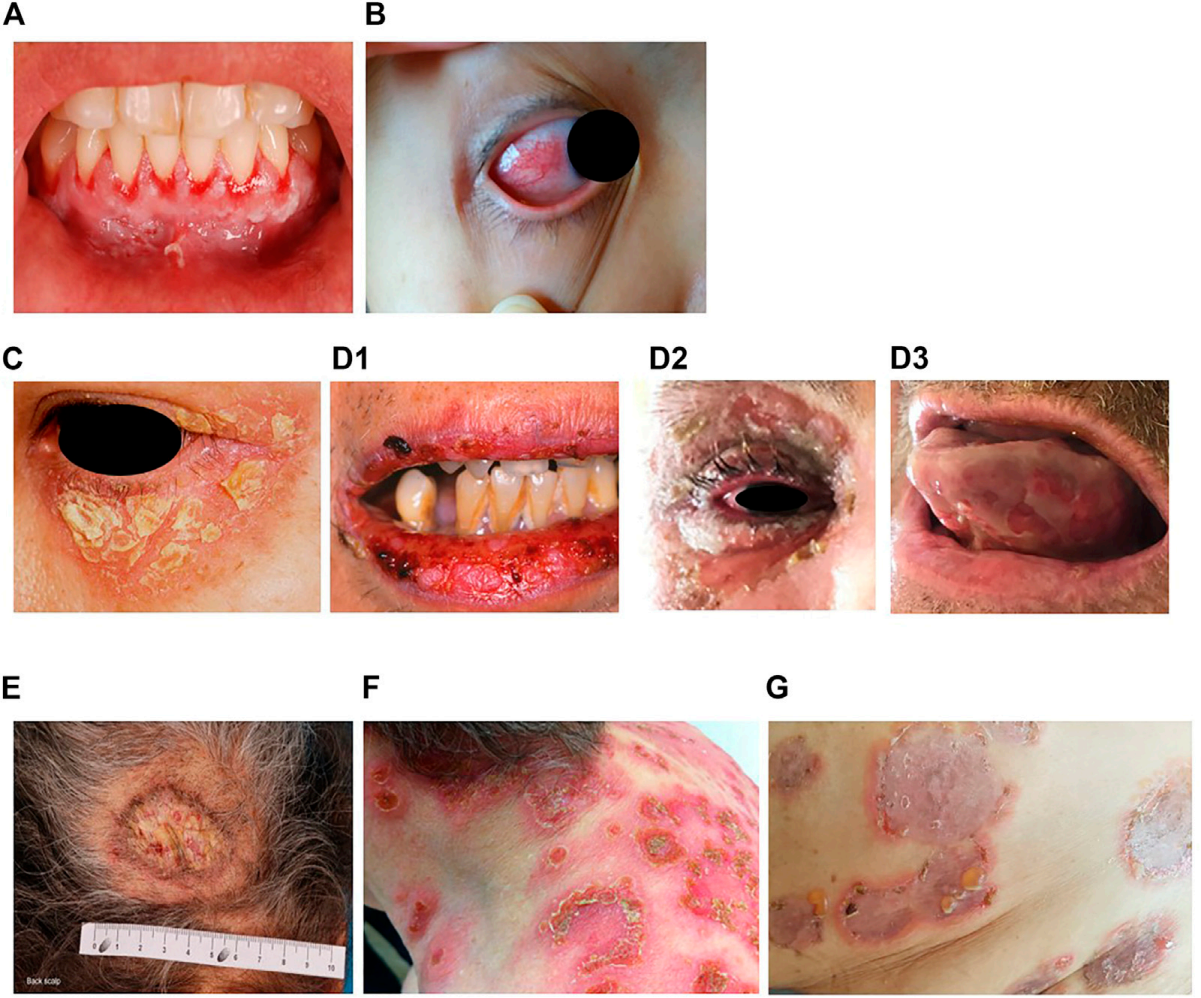
Erosions of the alvealor gingiva and attached mucosa **(A)** and conjuctival involvement with episcleritis **(B)** in patients with pemphigus vulgaris. Puff pastry-like scales and crusts on the periorbital skin of a patient with pemphigus foliaceus **(C)**. Severe stomatitis crusting and bleeding erosions on the lips that extended beyond the vermilion **(D1)**, severe pseudomembranous conjuctivitis with mucus discharge and eyelid erosions **(D2)**, diffuse lingual erotions **(D3)** in patient with paraneoplastics pemphigus. Vegetative plaque of pemphigus vegetans on the patient’s occipital scalp **(E)**. Post-pustular crusts with rounded or annular disposition in the trunk and neck in a patient with profuse IgA pemphigus **(F)**. Trunk lesions with annular lesions in a pemphigus herpetiformis patient **(G)**.

Pemphigus vulgaris (PV) (“*vulgus*” *which* means “common” in Latin) accounts for up to 70% of pemphigus cases (Joly and Litrowski, 2011; Jelti et al., 2019) with an estimated incidence of between one and five cases per million inhabitants per year (Bastuji-Garin et al., 1995, 1996; Jelti et al., 2019). However, incidence varies greatly between different regions and ethnicities: while PV occurs at a rate of less than 0.76 per million population in Finland or less than 0.5 per million population in Germany (Hübner et al., 2016), its incidence is more than twenty times higher in the United States and Israel (Hammers and Stanley, 2016; Kridin, 2018). Regardless of the population, PV typically affects adults between the age of 45 and 65 years, with a slight female predominance (Joly and Litrowski, 2011; Didona et al., 2019). PV cases are extremely rare in children and its incidence increases with age (Kneisel and Hertl, 2011b). Almost all PV cases will develop oral mucosal lesions during the course of the disease; oral mucosal lesions being the first site involved in more 50–70% (Shamim et al., 2008; Pollmann et al., 2018) with lesions affecting the gingiva, palate, floor of the mouth, buccal mucosa, lips and tongue (Kneisel and Hertl, 2011a). Blistering in these sites can cause symptoms ranging from mild discomfort to such severe pain during eating that rapid weight loss ensues. Other mucous membranes may also be involved, including the nasal, pharyngeal, laryngo-oesophageal, urethral, genital (glans penis, vulva), perianal and conjunctival mucosae (Bystryn and Rudolph, 2005; Kneisel and Hertl, 2011a) (Figures 1A,B).

In muco-cutaneous variants, non-scarring skin lesions (flaccid blisters, crusts, erosions) arise during the course of the disease. Although any parts of the body can be affected, the predilection sites of PV lesions encompass face, neck, axillary folds, scalp and trunk, notably in seborrheic areas or exposed to mechanical stress. Recent studies have also revealed other manifestations, such as nail involvement in patients with extensive disease (Pietkiewicz et al., 2018; De et al., 2019), and post-inflammatory hyperpigmentation that can last for months in patients with darker skin (Tamazian and Simpson, 2020). In case of cutaneous involvement, erosions can be induced in perilesional skin by applying tangential pressure, also known as Nikolsky’s sign. The diagnosis of pemphigus is evoked upon clinical examination; histological analysis of a skin/mucous membrane biopsy taken in a lesional area and showing acantholysis is required to support the diagnosis. Direct immunofluorescence examination of biopsy samples taken in perilesional areas is considered the “gold standard” to confirm PV diagnosis, revealing intercellular deposits of IgG and/or C3 on the surface of keratinocytes in the epidermis or mucosa (Harman et al., 2017; Joly et al., 2020), but with the disadvantage of requiring invasive sampling. Additional immunological tests are carried out in parallel, including detection of autoantibodies in serum targeting the epithelial cell by indirect immunofluorescence (IIF) on monkey oesophagus or human skin, and by enzyme-linked immunosorbent assays (ELISA) to detect circulating anti-desmoglein autoantibodies surface (anti-Dsg3 alone where there is mucosal involvement only, or anti-Dsg3 and anti-Dsg 1 in patients with mucocutaneous disease) (Joly et al., 2020). Of note, anti-Dsg ELISA is positive in 95% of cases and anti-Dsg values correlate with disease activity (Joly et al., 2020).

### Pemphigus Foliaceus

Pemphigus foliaceus (PF) (“*folium*” meaning “leaf” in Latin) is the second most common type of pemphigus and accounts for 27% of cases in France (Gonçalves et al., 2011; Jelti et al., 2019). Unlike PV, PF overall affects a broader age range of individuals due to the varied age of onset of several PF sub-variants (Chapman et al., 2018). PF-associated acantholysis occurs within the subcorneal layer of the epidermis, leading to more flaccid and fragile blisters that involve the skin and spare the mucous membranes (Joly and Litrowski, 2011). PF blistering and erosions typically exhibit “puff pastry-like” scales and crusts which tend to affect the seborrheic areas on the body: the scalp, face, presternal and interscapular skin (Figure 1C). In severe forms, PF might present as an erythroderma (chronic erythema involving more than 90% of the body’s surface area), in contrast with the large erosive areas found in PV. Subcorneal pustules or acantholytic clefts in the granular layer of the epidermis are distinguishing histological features of PF, which are important because both direct and indirect immunofluorescence findings are similar to those seen in PV. However, only anti-Dsg1 antibodies are present, accounting for the absence of mucosal lesions in PF (see Dsg 1/Dsg 3 compensation theory, below, and Mahoney et al. (1999)).

Several clinical sub-variants of PF which have been characterised have interesting and distinctive features. Notably, pemphigus erythematosus (PE) (also known as Senear-Usher syndrome) has a milder presentation with lesions often confined to or first seen in the malar area of the face in a butterfly shape, as typically found in lupus erythematosus (Amerian and Ahmed, 1985; Khachemoune et al., 2006; Hobbs et al., 2021). More extensive involvement might be seen in photo-distributed areas. Patients with PE also present with laboratory findings suggestive of systemic lupus erythematosus, including the presence of anti-nuclear antibodies in 30–80% of cases (Malik and Ahmed, 2007; Oktarina et al., 2012; Pérez-Pérez et al., 2012). It is not yet known how or whether these antibodies are related to the pathology seen in PE.

In addition to PE, three PF sub-variants restricted to particular locations or populations have been described. The first such “endemic” form to be described, in 1903, was fogo selvagem, which is found in subtropical areas of Brazil (Diaz et al., 1989; Aoki et al., 2015), where its prevalence can be as high as 3–5% of inhabitants (Gonçalves et al., 2011). While PF in children is generally rare (Kanwar and Kaur, 1991; Mintz and Morel, 2011), up to 30% of those affected by fogo selvagem are children or young adults (Diaz et al., 1989; Aoki et al., 2015). Colombian pemphigus foliaceus is a PF sub-variant that occurs within the El Bagre area of Northern Colombia (Abrèu-Velez et al., 2003a; Abrèu-Velez et al., 2003b), and closely resembles fogo selvagem, except for its male preponderance (Abrèu-Velez et al., 2003b). The third endemic form is found in north African Arab countries, mostly in the southern area of Tunisia but also in Algeria and Morocco (Morini et al., 1993; Abida et al., 2009), and some sub-Saharan countries, such as Mali (Mahé et al., 1996). A predominance of cases among young women aged 25 to 34 years had been described in Tunisia (Bastuji-Garin et al., 1995, 1996) and familial cases have also been reported (Morini et al., 1993). The geographical restriction, the age and the more extensive lesions of these sub-variants pose intriguing questions on the aetiology of the disease, which in some cases has been partially resolved (see below).

### Paraneoplastic Pemphigus

Paraneoplastic pemphigus (PNP) was first recognised in 1990 (Anhalt et al., 1990), and represents about 5% of pemphigus cases (Paolino et al., 2017). PNP is seen almost exclusively in patients with co-occurring neoplasms including lymphoproliferative disorders such as Castleman’s disease and non-Hodgkin lymphoma, as well as thymoma, chronic lymphocytic leukaemia, and various carcinomas and sarcomas (Anhalt et al., 1990; Joly et al., 2000; Leger et al., 2012; Ohzono et al., 2015; Yatim et al., 2019). PNP predominantly affects patients between 45 and 70 years of age, but may also occur in children, particularly when associated with Castleman’s disease (Wieczorek and Czernik, 2016). The condition typically presents as PV with severe mucocutaneous lesions, often with erosions and ulcers involving the tongue, and lesions on the lips that frequently extend to the vermillion border (Yatim et al., 2019). Other mucous membrane, such nasal, conjunctival and genital mucosa, may also be involved (Yatim et al., 2019): alongside, PNP-suggestive features of skin involvement are polymorphic, with pustular, erythema multiform-like or lichenoid lesions in addition to the classical flaccid bullous form and typical erosions (Anhalt, 2004; Leger et al., 2012; Ohzono et al., 2015) (Figures 1D1–D3). PNP may also affect internal organs, particularly the lung, and, more rarely, the gastrointestinal mucosa (Odani et al., 2020). The severity of PNP, possibility of multi-organ involvement, and co-occurrence with underlying malignancy together combine to give a high mortality rate (Leger et al., 2012; Ohzono et al., 2015; Wieczorek and Czernik, 2016; Jelti et al., 2019).

Histologically, PNP is characterized by a “tombstone” appearance of acantholytic basal keratinocytes, associated with an interface or lichenoid dermatitis caused by self-reactive CD4^+^ and CD8^+^ T cells, and with epidermal exocytosis and dyskeratotic or necrotic keratinocytes (Joly et al., 2020). Linear IgG/C3 deposits at the dermal-epidermal junction might also be present, in addition to positive intercellular labelling of the epithelium by DIF (Joly et al., 2020). Patients with PNP may develop IgG autoantibodies against a diverse array of antigens, including: Dsg3 (Amagai et al., 1998; Brandt et al., 2012), and several members of the plakin family (Mahoney et al., 1998) including the desmosomal plaque proteins desmoplakin I and II (Hashimoto et al., 1995), envoplakin (Kim et al., 1997), periplakin (Mahoney et al., 1998; Kazerounian et al., 2000), as well as against hemi-desmosomal adhesion molecules such as plectin and bullous pemphigoid antigen (BP) 230 (also known as dystonin-e), BP180 (also known as collagen α-1[XVII] chain) (Maier et al., 2017) and the proteinase inhibitor α2-macroglobulin-like protein 1 (Schepens et al., 2010). In addition, approximately 75% of patients exhibit antibodies against desmocollins (Dsc) 1, 2 or 3 (Ishii et al., 2015; Ohzono et al., 2015). Anti-epiplakin antibodies are especially significant, both clinically and diagnostically as they are thought to be responsible for the occurrence of often-fatal bronchiolitis obliterans, which results from loss of adhesion of the lung epithelium (Tsuchisaka et al., 2016; Tsuchisaka et al., 2016). Considering these immunological features of PNP, IIF examination of the serum is usually performed using rat bladder to investigate reactivity against plakin proteins (Joly et al., 2000). ELISA for anti-Dsg and anti-envoplakin antibodies are usually made with immunoblots with epidermal extracts or extracts from cultured keratinocytes to investigate IgG reactivity against other antigens, especially anti-plakins (Probst et al., 2009; Joly et al., 2020). In the case of PNP, co-morbid malignancy is clear, and for some time the mechanism of association has been known. The emergence of PNP appears to be related to the generation of antibodies against tumour antigens which cross-react with epithelial antigens, including both desmosomal and hemi-desmosomal proteins. This process is similarly seen in the case of some environmental antigens (see below) in endemic PV, but similar “trigger antigens” have yet to be investigated or defined in other pemphigus variants.

### Pemphigus Vegetans

Pemphigus vegetans, a rare clinical variant of PV which represents 1–2% of all pemphigus cases (von Köckritz et al., 2017). Due to it’s rarity, only few papers in the literature are specifically devoted to it with most of them are case reports (Mergler et al., 2017; Cuellar et al., 2020; Liu et al., 2020; Verma et al., 2020). Pemphigus vegetans is characterized by hypertrophic papillomatous vegetative skin lesions that preferentially affect intertriginous sites, such as the axillae, inframammary folds, umbilicus, inguinal creases, anogenital skin, scalp, and neck (Jain et al., 1989; Huang et al., 2005; Cozzani et al., 2007) (Figure 1E). Depending on the morphology of the initial lesions and the clinical course, pemphigus vegetans is further subdivided into two types: Neumann and Hallopeau. The Neumann type presents with flaccid blisters and erosions, evolving into fetid-odoured, whitish, macerated, hypertrophic, papillomatous plaques in the intertriginous body folds and lips, and more rarely on the trunk and extremities; the oral mucosa is usually involved (Leroy et al., 1982; Ahmed and Blose, 1984). By contrast, the Hallopeau type is associated with localised, pustular lesions that rupture and coalesce into vegetative erosions, and does not exhibit oral mucosa involvement (Zaraa et al., 2011; Imchen et al., 2014). Autoantibodies in pemphigus vegetans are against Dsg1 and Dsg3, as well as other proteins constituting the desmosome (Hashimoto et al., 1994; Cozzani et al., 2007).

### IgA Pemphigus

IgA pemphigus, even rarer among the pemphigus family, is characterized clinically by annular erosive plaques rimmed by pustules and affects the skin of the trunk, proximal extremities, and intertriginous regions (Figure 1F) (Geller et al., 2014). Mucosal involvement is uncommon (Kridin et al., 2020) and acantholysis is usually absent (Tsuruta et al., 2011). Here, IgA autoantibodies recognising various dermosomal proteins bind the epidermal cell surface and are detectable by direct immunofluorescence; while immune-serological assays are used to detect circulating IgA autoantibodies in these patients (Nishikawa et al., 1991; Geller et al., 2014). Depending on the level of pustule formation, IgA pemphigus can be divided into two major clinical and histological subtypes: intraepidermal neutrophilic dermatosis (IEN), which is characterized by suprabasilar pustules located at the lower or entire epidermis and associated with the presence of IgA antibodies against Dsg1 or Dsg3, and other yet-unidentified antigens; and subcorneal pustular dermatosis (SPD), in which patients exhibit subcorneal pustules in the upper epidermis and auto-reactive IgA binds desmocollin 1 (Dsc1) (Hashimoto et al., 1997; Karpati et al., 2000; Yasuda et al., 2000). IgA pemphigus may also be associated with IgA gammopathies, haematological disorders and ulcerative colitis (Hashimoto et al., 1997; Kridin et al., 2020).

### Neonatal Pemphigus Vulgaris

Neonatal pemphigus vulgaris (NPV) is a transient immunobullous disease induced by transplacental transfer of maternal autoantibodies from a mother with PV to her newborn (Fenniche et al., 2006; Gushi et al., 2008; Turrentine et al., 2014; Carvalho et al., 2019; Fenner et al., 2020; Foster et al., 2021). First reported in 1975 (Salihbegović-Opalić et al., 1975; Zhao et al., 2016), few cases exist in the literature, as female patients with pemphigus can experience infertility, premature delivery and stillbirth (Ouahes et al., 1997). NPV has various clinical manifestations, including skin and mucosa defects, blisters and erosions at birth, and extensive skin exfoliation which can arise early after birth. As maternal-derived antibody levels drop, NPV resolves spontaneously or with mild topical corticosteroid treatment within 4 weeks of birth (Kardos et al., 2009). Importantly, before the development of the neonatal mouse model of PV, clinical observation of NPV gave the first evidence of the direct pathogenicity of pemphigus autoantibodies.

### Pemphigus Herpetiformis

Pemphigus herpetiformis (PH), first named in 1975 (Jablonska et al., 1975), is a rare subtype of pemphigus. It clinically resembles to dermatitis herpetiformis, with patients exhibiting pruritic herpetiform blisters, and/or annular urticarial plaques with or without erosions, or scaly erosive patches mimicking eczema (Tay et al., 2018) (Figure 1G). However, immunological findings are consistent with pemphigus (Kasperkiewicz et al., 2014; Costa et al., 2019). Direct and indirect immunofluorescence demonstrate intercellular deposits of IgG and/or C3, with Dsg as the main target of autoantibodies, with additional recognition of Dsc (1,2 3) and other hemidesmosomal antigens, seen in some patients (Kozlowska et al., 2003; Ohata et al., 2013; Ueda et al., 2013; On et al., 2015). The histology of PH is less specific than that of PV and ranges from intraepidermal eosinophils or neutrophilic spongiosis (Hashimoto et al., 1983) to intraepidermal vesicles filled with neutrophils or eosinophils, and may also encompass dermal papillary micro-abscesses (Costa et al., 2019).

## The Pathway to Pemphigus: Risk Factors for Developing the Disease

Many autoimmune diseases have a complex aetiology, and pemphigus is no exception. Factors as diverse as genetics, environment, pre-existing health conditions, medication use and even post-vaccine reactions have been linked with the emergence of pemphigus. In this section we will review some of the most recent observations that are building up the picture of circumstances and pathways leading to the clinical manifestation of pemphigus.

### Genetic Risk Factors

The familial clustering of certain variants of pemphigus, combined with its predilection for some ethnicities and co-occurrence of other autoimmune diseases in patients, all point towards a significant genetic component to susceptibility. To date, the strongest link has been reported for the association between PV and HLA class II genes (Gazit and Loewenthal, 2005; Tron et al., 2006; Vodo et al., 2018). Studies have shown that HLA alleles DRB1*04:02 and DQB1*05:03 represent the most common PV associated alleles (Ahmed et al., 1991; Carcassi et al., 1996; Delgado et al., 1996; Lee et al., 1998; Lombardi et al., 1999; Loiseau et al., 2000; Miyagawa et al., 2002; Geng et al., 2005; Liu et al., 2008; Shams et al., 2009; Tunca et al., 2010; Párnická et al., 2013; Brochado et al., 2016), with the majority of the patients with PV expressing one of these two alleles. While some of the HLA types are more population-specific, there are others that are associated with PV across numerous ethnic groups: a link between HLA-DRB1*04:02 and DQB1*03:02 with PV in the Jewish population has been highlighted (Gazit and Loewenthal, 2005), while HLA-DQB1*05:03 was found in association with PV in non-Jewish populations (Ahmed et al., 1991). Studies in Han Chinese patients with PV have highlighted DRB1*04, DRB1*14 and DQB1*05:03 as relevant risk alleles (Zhou et al., 2003; Geng et al., 2005; Liu et al., 2008; Gao et al., 2018), as well as HLA-DRB1*03 and HLA-CW*14 in the same Chinese population with PNP (Martel et al., 2003; Liu et al., 2008). Meta-analyses of the correlation between PV occurrence and HLA-DRB1 and HLA-DQB1 have shown that DRB1*04, DRB1*08, DRB1*14, DQB1*03:02 and DQB1*05:03 are significant susceptibility factors for PV while DRB1*03, DRB1*07, DRB1*15, DQB1*02, DQB1* 03:03, DQB1*05:01 and DQB1*06:01 were less common in patients with PV compared to healthy individuals (Yan et al., 2012; Li et al., 2018). In addition to HLA class II alleles, there may be some association between PV and certain HLA class I alleles within specific ethnic groups, for example, HLA-A10 and -B15 in the Japanese population (Hashimoto et al., 1977; Miyagawa et al., 2002), and HLA-A3, -A26, and -B60 in the Han Chinese population (Gao et al., 2018). However, although numerous studies have suggested that HLA status could be a key driver of pemphigus disease activity, the mechanistic link between the HLA genetic profile and the clinical picture within patients remains unclear.

Alongside the HLA susceptibility studies, other research has uncovered associations between PV and autoantigen or immune gene sequences: specific *Dsg3* haplotypes were significantly linked with PV in both British and Indian cohorts (Capon et al., 2006); while single nucleotide polymorphisms within the variable region of the immunoglobulin heavy chain *VH3* gene were associated with PF in two patients (Yamagami et al., 2009). Genetic variants of the cytokine genes *TNF-α*, *IL-6* and *IL-10* gene have also been linked with PV (Torzecka et al., 2003; Pereira et al., 2004; Eberhard et al., 2005; Mosaad et al., 2012; Toumi et al., 2013; Dar et al., 2016; Vodo et al., 2016); as have those affecting the *TAP2* gene, which encodes a protein involved in peptide assembly and transport to HLA class I (Niizeki et al., 2004; Slomov et al., 2005). Immune-associated SNPs that have been linked with pemphigus include those within the *ST18* gene, which encodes a transcription factor involved in inflammation and apoptosis (Sarig et al., 2012; Vodo et al., 2016; Etesami et al., 2018; De Bonis et al., 2019; Radeva et al., 2019; Assaf et al., 2021); and those within the *CTLA4* and *CD86* genes, whose protein products are expressed on antigen-presenting cells (APC) and are involved in activating T-cells and stimulating IgG production by B cells (Dalla-Costa et al., 2010; Tanasilovic et al., 2017).

While strong linkages have been found to exist between pemphigus and some of the above HLAs, the vast majority of individuals who carry the PV-associated HLA susceptibility alleles do not develop the disease. This prompted a group of researchers to discover a subset of PV-related differentially expressed genes which suggests a “protection” signature in genetically susceptible individuals from developing PV (Dey-Rao et al., 2013).

Knowing the genetic susceptibility in pemphigus may spur further research to better understand the interactions between various genetic susceptibility factors, the role of epigenetics and the functional effects of the identified genetic variants in disease-relevant cells, and hopefully, ultimately, their influence on response to treatment and severity of disease (Petzl-Erler, 2020).

### Environmental Risk Factors

The lived-in environment is a complex mixture of factors that act across time and space, interacting with genetic susceptibility to affect health outcomes. Studying this intricate web of influences has led to the identification of significant factors linked with the development of pemphigus in specific populations.

In the case of endemic pemphigus foliaceus in Brazil, the observation that many of the adults affected by fogo selvagem were outdoor workers living in conditions of poor hygiene and low-quality housing, built in forest areas adjacent to rivers and streams (Diaz et al., 1989; Aoki et al., 2015), led researchers to seek environmental factors causing the disease. These investigations resulted in the identification of sand flies as a triggering factor the salivary protein LJM11 of *Lutzomyia longipalpis* (also known as black fly) (Culton et al., 2008) cross reacts with Dsg1 (Warren et al., 2000; Qian et al., 2012).

Ultraviolet radiation has been reported to exacerbate or cause new-onset of pemphigus (Fryer and Lebwohl, 1994; Muramatsu et al., 1996; Aghassi, 1998; Kano et al., 2000; Makino et al., 2014). A study using medical records review showed that the majority of pemphigus patients had the first manifestation of their disease in spring and summer of Sofia, Bulgaria (Tsankov et al., 2000). Hospitalisation primarily for pemphigus was also noted to be higher in days with higher UV index for Hispanic pemphigus patients in United States (Ren et al., 2019). In a more recent study which looked at seasonal patterns and triggering factors in non-endemic pemphigus foliaceous in Turkey (Sayar and Küçükoğlu, 2021), it was also observed that new onset and relapses of non-endemic pemphigus foliaceous occurred more often during the spring-summer season where UV radiation is at its peak, further supporting the possible role of UV radiation as a trigger factor in this disease. While it has been demonstrated that UVB induces acantholysis and epidermal intercellular deposition of immunoreactants in Fogo selvagem patients (Reis et al., 2000), the molecular pathway still needs to be fully elucidated.

Other environmental risk factors for the development of pemphigus include trauma (Daneshpazhooh et al., 2016), stress (Cremniter et al., 1998; Morell-Dubois et al., 2008), diet (Ruocco et al., 2001). Interestingly, smoking seems to confer a protective effect on pemphigus (Valikhani et al., 2007). Cigarette smoking has also been found to improve pemphigus disease and those who smoked had earlier disease remission (Valikhani et al., 2008). It is postulated that interaction of nicotine and other nicotinic agonists with nicotinic acetylcholine receptors on keratinocytes may promote cell–cell adherence (Grando and Dahl, 2000).

### Therapeutic Risk Factors

Drugs have been reported to trigger pemphigus. The three main groups of drugs which has been reported to be associated with new onset or exacerbation of pemphigus are the thiol drugs, phenol drugs and non-thiol/non-phenol drugs. Thiol drugs have sulfhydryl (-SH) group in their chemical structure and are probably the better studied group of drugs in the pathogenesis of pemphigus. In a systematic review of 170 drug-induced pemphigus (Ghaedi et al., 2021), penicillamine, captopril and bucillamine, which belong to the thiol drugs, were the most commonly reported drugs to induce pemphigus.

Biological thiol substances such as cysteine and glutathione has been shown to induce acantholysis in human skin fragments under certain experimental conditions (Ruocco et al., 1982). *In vitro* experiments subsequently demonstrated that thiol drugs (d-penicillamine, captopril, thiopronine and piroxicam) induce acantholytic splitting in human skin fragments or skin cultures in the absence of pemphigus antibodies (i.e biochemical acantholysis) (Ruocco et al., 1993). It is postulated that thiol drugs form drug-cysteine disulfides which directly interfere with desmoglein adhesions between keratinocytes, or indirectly through antigen modification and autoantibodies production (Wolf and Ruocco, 1997). Plasminogen activator inhibitor, a proteolytic enzyme, has been shown to be important in preventing immunoglobulin-induced acantholysis in pemphigus (Hashimoto et al., 1989). Thiol drugs, on the other hand, stimulate plasminogen activator through inhibition of their natural inhibitors, leading to promotion of acantholysis (Hashimoto et al., 1984; Lombardi et al., 1993).

Other than drugs, vaccines have also been reported to trigger or exacerbate pemphigus. Amongst them are influenza (Mignogna et al., 2000; De Simone et al., 2008), hepatitis B (Berkun et al., 2005), rabies (Yalçin and Alli, 2007), tetanus (Cozzani et al., 2002) and more recently, SARS-COV2 vaccines (Damiani et al., 2021; Koutlas et al., 2021; Lua et al., 2021; Solimani et al., 2021; Thongprasom et al., 2021). While the mechanisms behind vaccine-induced pemphigus has not been worked out, there are several hypotheses other than those proposed for drug-induced pemphigus. These include autoimmunity arising from molecular mimicry of vaccines or their adjuvants with self-antigens (Shoenfeld et al., 2000), and non-specific activation of innate immunity and expansion of autoreactive T cells (Horwitz and Sarvetnick, 1999). It is however interesting to note that infections which these vaccines are used against, have not been reported as triggers for pemphigus (Brenner et al., 2002).

## Autoimmune Pathways and Processes in Pemphigus

### Autoantibodies

Following the identification of Dsg 1 and 3 as critical pemphigus autoantigens (Eyre and Stanley, 1987, 1988; Hashimoto et al., 1990; Amagai et al., 1991; Mahoney et al., 1999; Amagai and Stanley, 2012), many studies have investigated the autoantibodies and autoreactive B cells that drive pemphigus pathogenesis. The extracellular domains, EC1 and EC2, of Dsg have been defined as the main target region recognised by autoantibodies from pemphigus patients (Amagai et al., 1992, 1995; Futei et al., 2000; Sekiguchi et al., 2001; Payne et al., 2005; Ishii et al., 2008; Chan et al., 2010; Di Zenzo et al., 2012), with some evidence of minor EC4 recognition, and IgG4 is now known to be the major pathogenic antibody isotype (Dmochowski et al., 1992; Allen et al., 1993; Wilson et al., 1993). However, other isotypes may also be pathogenic in some variants or circumstances (Payne et al., 2005; Saleh et al., 2015). Interesting new data have revealed that DSG3-specific memory B cells have an activated phenotype and show signs of ongoing affinity maturation and clonal selection (Cho et al., 2019).

Although Dsg-specific autoantibody titre was long-thought to correlate with disease activity in pemphigus (Amagai et al., 1999; Daneshpazhooh et al., 2007; Abasq et al., 2009; Schmidt et al., 2010), more recent studies have detected anti-Dsg3 IgG antibodies in patients who were in clinical remission, suggesting the existence of non-pathogenic Dsg-specific IgG (Belloni-Fortina et al., 2009; Yoshida et al., 2017; Chernyavsky et al., 2019). This concept of pathogenicity has been confirmed by *in vivo* and *in vitro* studies demonstrating that pathogenic IgG autoantibodies preferentially target the NH2-terminal portion of Dsg3. Non-pathogenic autoantibodies recognize epitopes of the membrane proximal COOH-terminus of the Dsg ectodomains (Amagai et al., 1992; Futei et al., 2000; Bhol and Razzaque Ahmed, 2002; Tsunoda et al., 2003; Chan et al., 2010; Lo et al., 2016). Comparing conventional and EDTA-pretreated anti-Dsg3 commercial ELISA, Kamiya et al. demonstrated the presence of anti-Dsg antibodies directed against calcium-dependent epitopes targeting the extracellular domains of Dsg3 (Kamiya et al., 2012) and found a higher correlation between antibodies against calcium-dependent epitopes of Dsg3 and clinical activity than with total anti-Dsg3 antibodies (Kamiya et al., 2013). The pathogenic activity of these IgG directed against Ca-dependent epitope is sustained by their ability to induce the keratinocyte dissociation *in vivo* (Kamiya et al., 2012).

In PF and PV subtypes, Dsg1 and Dsg3 specific antibodies might directly affect the clinical presentation due to their differential expression in skin and mucous membranes. While patients with PV often experience mucosal and cutaneous blistering, and possess both anti-Dsg1 and anti-Dsg3 antibodies, those with PF have antibodies only to Dsg1 and present with cutaneous but not mucosal involvement. Dsg1 is mainly expressed in the upper epidermis and upper layers of the mucosa, while Dsg3 is predominantly expressed in the suprabasal layers of the epidermis and throughout the mucosal epithelium. This differential expression of Dsg1 and Dsg3 in the epidermis and mucosa together with the postulation that the Dsg can compensate for each other’s adhesive function when expressed on the same keratinocyte: this is the Dsg1/Dsg3 compensation theory (Amagai, 2009; Amagai and Stanley, 2012). Unfortunately, this concept cannot explain all clinical forms of pemphigus, especially those with atypical features, and the factors affecting the ability or extent of functional compensation have yet to be revealed. With better immunological profiling of pemphigus, a modification of the compensation theory and new alternative pathogenic mechanisms need to be evaluated (Koga et al., 2012; Sardana et al., 2013; Carew and Wagner, 2014; Ahmed et al., 2016; Öktem et al., 2018).

**FIGURE 2:**
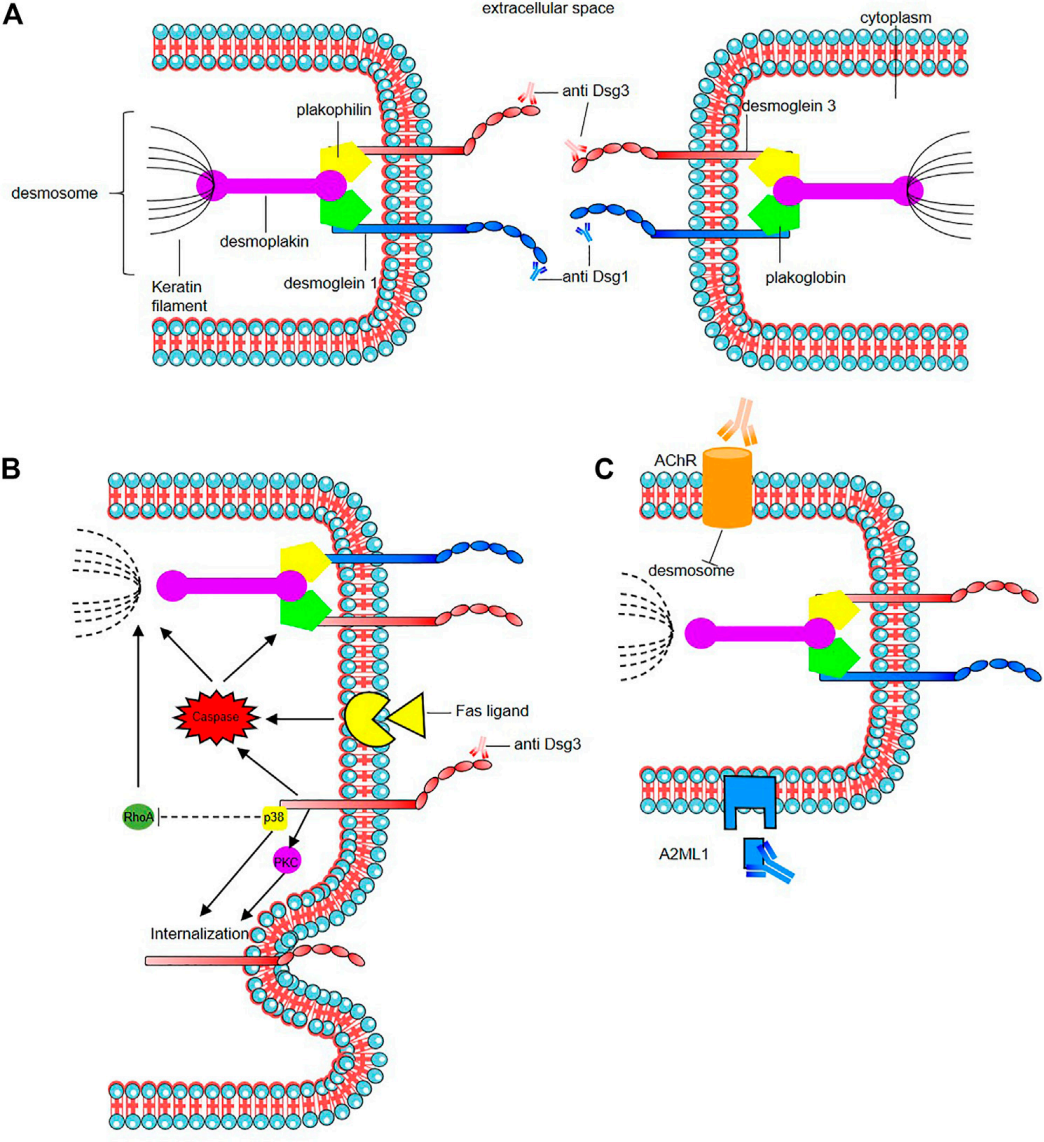
Mode of action of the pathogenic autoantibodies in pemphigus. **(A)** Interaction of pathogenic autoantibodies the NH2-terminal of desmosomal protien leading to steric hindrance of trans-Dsg binding. **(B)** Binding of autoantibodies to desmoglein induce an alteration of cellular signaling affecting components of multiple pathways including p38 MAPK (p38), protien kinase C (PKC) including an internalization of Dsg3. The autoantibodies binding reduce the RhoA activity in the p38MPAK-dependent manner which impact the reorganization of the actin cytoskeleton and drive further loss of desmosomal integrity. Caspace signaling may be activated by signal induced by the binding of autoantibodies to desmoglein by the Fas ligand/receptor pathway including pre-apoptotic caspase signaling. **(C)** Anti-keratinocyte α-acetycholine receptor (AChR) IgG autoantibodies induce signal causing disassembly of desmosomes, leading to acantholysis and blistering. In PNP, autoantibody binding also seems to inhibit A2ML1 (α-2 microglobulin-like 1) impacting the activation of a protease inhibitor. For a recent in-depth review see Schmitt and Waschke (2021).

The mode of action of the pathogenic autoantibodies found in pemphigus patients is now also well-defined (Figure 2). The desmosomal depletion that leads to acantholysis can be divided into two major mechanisms: firstly, the interaction of IgG autoantibodies with the NH2-terminal EC1 subdomains of Dsg1 and Dsg3 in PF and PV, respectively, leads to steric hindrance of trans-Dsg binding (Heupel et al., 2008; Jennings et al., 2011; Saito et al., 2012), and also disrupts the interaction between Dsg and flotillin (Völlner et al., 2016), together leading to the loss of desmosomal integrity (Figure 2A). The second mechanism is an alteration to cellular signalling that is induced by autoantibody binding affecting components of multiple pathways including p38 MAPK, protein kinase C (PKC), c-Jun N-terminal kinases (JNK), RhoA and caspases 3, 6, 8 and 9, which leads to Dsg endocytosis and depletion driving further loss of desmosomal integrity and adhesion (Puviani et al., 2003; Berkowitz et al., 2005, 2006, 2008; Frušić-Zlotkin et al., 2005; Kawasaki et al., 2006; Lee et al., 2009; Cirillo et al., 2010; Mao et al., 2011) (Figure 2B). In PNP, autoantibody binding also seems to inhibit A2ML1 (α-2 microglobulin-like 1) impacting the activation of a protease inhibitor (Inaoki et al., 2001; Schepens et al., 2010; Numata et al., 2013) (Figure 2C). For a recent in-depth review of this topic see (Schmitt and Waschke, 2021).

In addition to anti-Dsg IgG autoantibodies, various antibodies targeting other autoantigens have been identified, notably with proteomics studies (Kalantari-Dehaghi et al., 2013), in the sera of a minority of patients with various forms of pemphigus with an unclear pathogenic role (Nguyen et al., 2000b; Ahmed et al., 2016; Amber et al., 2018). IgG and IgA autoantibodies against desmocollins (Dsc1, Dsc2 and Dsc3 being Ca^2^+-dependent cadherins involved in desmosome assembly) have been detected in variable percentage of pemphigus patients (5–60%), according cohorts, Dsc subfamily and the type of pemphigus variant (Dmochowski et al., 1993; Müller et al., 2009; Ishii et al., 2015; Mindorf et al., 2017). Their rates are higher in rare variants such as IgA pemphigus, PNP, and pemphigus herpetiformis (Hashimoto et al., 1997; Müller et al., 2009; Ishii et al., 2015; Mindorf et al., 2017). It has been demonstrated that anti-Dsc3 react against extra cellular domains of Dsc3 in human skin model resulting in intraepidermal blister formation, and in loss of intercellular cohesion in keratinocyte culture induced by targeting of the Dsc3/Dsg1 binding on keratinocyte cell surface as Dsc and Dsg are interacting by homophilic and heterophilic trans-interaction (Spindler et al., 2009). Anti-keratinocyte α-acetylcholine receptor (AChR) IgG autoantibodies have been also reported in 85% of PV and PF patients (Nguyen et al., 1998; Lakshmi et al., 2017). Inhibition of these receptors which control keratinocyte adhesion and motility may induce signals causing disassembly of desmosomes, leading to acantholysis and blistering (Figure 2C). Autoantibodies against muscarinic AchRs but also against mitochondrial nAChR were found (Chernyavsky et al., 2015). A correlation of anti-muscarinic AchRs antibody titers and anti-Dsg antibody titers with the severity of disease in patients with pemphigus has been reported (Lakshmi et al., 2017). The role of these autoantibodies found in sera from pemphigus is unclear, as the fact that they could result from epitope spreading following the loss of tolerance or have a synergistic action with anti-Dsg in pemphigus pathogenesis (Nguyen et al., 2000a; Grando, 2000). Some react mainly against structural components of adhesion molecules such as desmosomes like autoantibodies against desmoplakin 1, desmoplakin 2 (Kim et al., 2001), Dsg4 (Kljuic et al., 2003; Nagasaka et al., 2004), E-cadherin (Evangelista et al., 2008; Oliveira et al., 2013), plakoglobin (Korman et al., 1989; Ishii et al., 2001), plakophilin 3 (Lambert et al., 2010), FCεR1 (Fiebiger et al., 1998), and pemphaxin (Nguyen et al., 2000a) while other impact protease inhibitors such as anti- A2ML1 or anti-hSPCA1 (Ca21/Mg21-ATPase encoded by ATP2C1 gene) which is involved in Golgi apparatus trafficking and of which impairment could lead to the impairment of proteic assembly and adress to the membrane as in hailey-hailey disease (Kalantari-Dehaghi et al., 2013). Although mouse models have been designed to incorporate some of these autoantibodies (Chen et al., 2008; Rafei et al., 2011), their role in pemphigus pathogenesis is still unclear (Spindler et al., 2018) and more complex mouse model need to be designed.

### T Cells

While autoantibodies produced by B cells are the key and direct pathogenic mediators of pemphigus, recent studies are beginning to illuminate the broader autoimmune picture, with an emphasis on the role of T cell subsets. The disruption of peripheral T cell-mediated tolerance to self-antigens can be a critical event leading to autoantibody generation, inflammation, and tissue infiltration by immune cells: thus, a role for autoreactive helper T cells in driving pemphigus has long been postulated. Patients with pemphigus possess Dsg3-reactive Th1 and Th2 cells (Lin et al., 1997; Rizzo et al., 2005), with some studies finding comparable frequencies of the two subsets (Eming et al., 2000), and others detecting greater numbers of autoreactive Th1 compared to Th2 cells (Veldman et al., 2003). Studies in a humanized HLA-DRB1*04:02 transgenic mouse model showed that T-lymphocytes recognize human desmoglein 3 epitopes in the context of HLA-DRB1*04:02 (Eming et al., 2014). This recognition was associated with a CD40-CD40L-dependent T-cell—B-cell interaction that lead to the induction of pathogenic IgG autoantibodies, which in turn triggered intraepidermal blister formation. Although it seems likely that both Dsg3-reactive Th1 and Th2 cell are important for pemphigus development, most studies have focused on the role of Th2 cells and shown them be critical for the development of the disease. Frequencies of Dsg3-reactive Th2 cells are significantly higher in patients than controls, and their levels are positively correlated with disease activity (Rizzo et al., 2005). In addition to Th2 cells, Th2 cytokine levels are significantly higher in the serum of patients with PV than controls, suggesting an imbalance between Dsg3-reactive Th2 and Th1 cells in the pathogenesis of PV (Satyam et al., 2009; Lee et al., 2017). However, the role of other T cell subsets in pemphigus pathogenesis remained unclear.

Th17 cells are identified by their specific expression of the transcription factor RORγt and have been implicated in the autoimmune pathology of a range of conditions including psoriasis (Fitch et al., 2007), ankylosing spondylitis (Taams et al., 2018) and, more recently, pemphigus (Xu et al., 2013; Timoteo et al., 2017; Jmaa et al., 2018). In patients with PV, the frequency of Th17 cells in the peripheral blood is significantly higher than in healthy controls, especially during the acute onset and active chronic stages (Holstein et al., 2021). Moreover, the levels of Th17-associated molecules (IL-17A and CCL20) in the serum, and the relative expression levels of RORγt, CCR6 and CCL20 is also relatively higher in patients with PV than in controls (Asothai et al., 2015). Th17 cells are also present in lesional skin (Arakawa et al., 2009): accordingly, a comparison of the expression profiles of lesional versus perilesional and healthy skin from a patient with pemphigus identified an IL-17A-dominated immune signature, with high expression of genes involved in the IL-17A signalling pathway (Holstein et al., 2021).

Alongside Th1, Th2 and Th17 cells are T follicular helper cells (Tfh): these are a subset of CD4^+^ T lymphocytes expressing the transcription factor BCL6, and CXCR5, which enable them to migrate into germinal centres where they are required for the selection of high-affinity antibody-producing B cells and the development of memory B cells (Morita et al., 2011; Craft, 2012). Tfh cells also express the surface receptors programmed cell death protein-1 (PD-1), inducible T cell costimulator, and CD40L, and secrete IL-21 to promote B cell growth, activation, immunoglobulin class-switching and differentiation into memory B cells and plasmablasts (Zotos et al., 2010). Early indications of a role for Tfh cells in pemphigus came from studies showing that the frequency of circulating Tfh cells (defined as CD4^+^CXCR5^+^ T cells) and plasma concentrations of IL-21 were both significantly higher in patients with PV compared to controls (Holstein et al., 2021); and identified autoreactive IL-21-secreting cells in 50% of patients with PV (Hennerici et al., 2016). In addition, frequencies of Th17 and Tfh17 cells in the blood of patients with pemphigus correlate with levels of Dsg-specific CD19^+^CD27^+^ memory B cells (Maho-Vaillant et al., 2021), while patients with acute pemphigus exhibit higher levels of Dsg3-autoreactive Tfh17 cells (Hennerici et al., 2016). Moreover, *in vitro* coculture experiments revealed that Tfh17 cells are primarily responsible for inducing Dsg-specific autoantibody production by B cells (Holstein et al., 2021). Leading on from this, targeting the inducible co-stimulator expressed on CXCR5^+^PD-1^+^ Tfh cells suppressed the progression of PV in a murine model (Kim et al., 2020). All these findings show that Tfh17 cells are critically involved in the pathogenesis of pemphigus and offer novel targets for therapeutic intervention.

A major cell type tasked with preventing autoimmunity in the body is regulatory T cells (Treg), which exhibit a TCRαβ^+^Foxp3^+^CD4^+^ phenotype and develop in the thymus (Sakaguchi et al., 1995; Peterson, 2012). Treg cells possess potent immune-suppressive activity and their main role is to control inflammation and immune responses to self-antigens. Various studies have demonstrated that the proportion of Treg cells in the blood of patients with PV is markedly lower than in healthy donors (Veldman et al., 2004). Similar trends are observed in patients with PV in the acute onset and remittent stages: frequencies of Th17 and Treg cell populations are inversely correlated, indicating that the balance of Th17/Treg cells is disrupted in PV (Xu et al., 2013; Asothai et al., 2015, 4). Similarly, a lower ratio of Dsg3-specific type 1 regulatory T cells (Tr1) to Th2 cells in patients with PV has been described (Veldman et al., 2006). *In vivo*, mouse models of PV have demonstrated that Tregs control anti-Dsg3 antibody production, and that adoptive transfer of Tregs or the depletion of endogenous Tregs suppresses and increases anti-Dsg3 antibody production, respectively (Yokoyama et al., 2011). Alongside, *in vitro* studies have shown that Dsg3-specific Tr1 cells secrete IL-10, TGF-β, and IL-5 upon autoantigen stimulation and inhibit the proliferation of Dsg3-responsive, auto-reactive helper T clones in an autoantigen- and cell-number- dependent manner (Veldman et al., 2004). This study suggests that Treg cells are involved in the maintenance and restoration of tolerance against autoantigens in PV. Recent work also suggests that Treg cells from patients with PV may have defective CCR4-CCL22 ligand interactions (Asothai et al., 2015), which could lead to reduced homing to the sites of skin lesions.

The skin also provides a niche for long term tissue-resident memory T (TRM) cells to accumulate, where they can mediate durable protective immunity but may also be involved in driving inflammatory diseases (Ho and Kupper, 2019). Recently, a study reported the over-representation of CD4^+^ TRM in the lesional skin of patients with pemphigus (Zou et al., 2021). These T follicular helper-like CD4^+^ TRM cells were thought to promote local autoantibody production, which could result in the formation and recurrence of lesions. Another study suggested that the long-lasting response to rituximab in pemphigus could rely on the decrease of Dsg-specific circulating T follicular helper cells, which correlates with a sustained depletion of IgG-switched memory autoreactive B cells, leading to the disappearance of anti-Dsg antibody-secreting cells (Maho-Vaillant et al., 2021). These results support the therapeutic targeting of Tfh-like TRM cells in pemphigus treatment, perhaps via IRF4 which might serve as potential therapeutic target (Zou et al., 2021). Understanding the changes in phenotype, functions, and interactions of these, and other, T cell subsets is key to understanding the pathogenesis of pemphigus and for the development of new targeted therapeutics.

### Myeloid Cells

The myeloid cell population represents a major compartment of the immune system and includes dendritic cells, macrophages, monocytes and neutrophils (Bassler et al., 2019). Dermal dendritic cells (cDC1, cDC2) and epidermal Langerhans cells (LCs) represent the specialized antigen presenting cells of the skin and the functional “bridge” between innate and adaptive immunity. They are considered as sentinels of the skin that defend against pathogens by controlling the innate immune cell wave infiltrating the area during infection (Del Fresno et al., 2018; Janela et al., 2019) as well as by shaping the B and T cell mediated immune response: accordingly, they are key players in the maintenance of tolerance. Despite this, little is known of the role of the myeloid compartment in PV pathogenesis.

The frequency of myeloid DCs in the blood of patients with pemphigus is increased, and these cells exhibit altered expression of DC-associated stimulatory (CD40 and CD80) and inhibitory (PSGL1 and ILT3) markers compared to healthy donors (Das et al., 2020). These observations suggest a potential role of DC dysregulation in the immunopathogenesis of pemphigus. A recent study demonstrated that high numbers of LCs are present in perilesional skin from patients with pemphigus (Das et al., 2020); while *in vitro* work showed that LCs were able to capture the epidermal antigen Dsg3 via langerin and to present this antigen to T cells (Kitashima et al., 2018). Interestingly, LCs express the IL-2 receptor complex, and disruption of IL-2 signalling in LCs inhibits LC-mediated regulatory T cell expansion *in vitro* (Kitashima et al., 2018). Therefore, LCs may be important mediators of peripheral tolerance against epidermal autoantigens via IL-2 signalling, though this has yet to be directly demonstrated in pemphigus.

Another population of cells that exhibits potentially significant changes in pemphigus is myeloid-derived suppressor cells (MDSCs). This heterogeneous group of immature myeloid cells incorporates granulocytic or polymorphonuclear-like MDSCs (PMN-MDSCs) and monocytic MDSCs (M-MDSCs) (Gabrilovich and Nagaraj, 2009), which inhibit T cell activity via several mechanisms (Nagaraj et al., 2010). A population of CD66b + CD11b + PMN-MDSCs is expanded in the peripheral blood of patients with active pemphigus (Oktem et al., 2021), but not those in remission (Neri et al., 2021). *In vitro*, these PMN-MDSCs can suppress allogeneic T-cell proliferation and exhibit high levels of expression of characteristic effector molecules such as arginase I and interleukin-10 (Neri et al., 2021). In addition, a correlation between MDSC frequency and Th2/Th1 cell ratio has been highlighted, which suggests a possible role of these cells as regulators of Th cell responses in pemphigus (Neri et al., 2021).

Plasmacytoid DCs (pDCs), which were long-thought to be part of myeloid lineage but in fact come from a lymphoid progenitor (Dress et al., 2019), could also have a possible role in pemphigus pathogenesis. pDCs seems to be recruited at very high level into the lesional skin of pemphigus patients (Ramadan et al., 2019). It is thus possible that pDCs might be involved in the initial mechanisms leading to autoantibody production, but this has yet to be formally investigated.

It has been demonstrated in autoimmune diseases such as lupus, rheumatoid arthritis and small vessel vasculitis that neutrophil extracellular traps (NETs) are linked with immune complexes and responsible for tissue inflammation and polyclonal activation of B-cell as well as memory B-cell activation (Yu and Su, 2013; Delgado-Rizo et al., 2017; Apel et al., 2018; O’Neil et al., 2019). As neutrophil can be found in the skin of pemphigus, a clinical trial aims to assess the effects on B-cell activation and the phenotypic changes in B-cell population from pemphigus patients after stimulation by NETs (NCT04117529). The understanding of the link between innate and adaptive immunity is essential for a direct targeting of the different actors of the diseases.

## Comprehension of the Pemphigus by Using *In Vivo* Model of the Disease

As discussed above, there are many aspects of the aetiology, pathogenesis and treatment of pemphigus that we have yet to fully understand: these diseases are rare–very rare in the case of some variants and sub-variants–which limits opportunities for clinical studies, and robust insights can be hard to glean from the type of mechanistic experiments that are possible using human cells *in vitro*. In the past three decades, experiments using different mouse models of pemphigus have led directly or indirectly to the identification of the dominant autoantigens, the discovery of autoreactive T cells and B cells and their roles in the production of pathogenic IgG autoantibodies. These findings represent the sum of multiple studies employing different *in vivo* models, each focused on specific aspects of the autoimmune cascade, from loss of immunological tolerance on the level of T and B cells to the pathogenic effects of autoantibodies upon binding to their target autoantigen.

The first mouse model of pemphigus was the passive transfer model, in which IgG from the sera of patients with PV is injected intraperitoneally into neonatal mice, resulting in the formation of cutaneous blisters and erosions and reproducing the clinical, histologic, ultrastructural and immunologic features of the disease (Anhalt et al., 1982). Studies in these mice provided the first evidence that PV IgG alone are pathogenic *in vivo*, and facilitated the dissection of the mechanism of blister formation (Schulze et al., 2012). Later, the need to study the mechanisms leading to the generation of pathogenic autoantibodies in PV drove the development of an active disease model: Dsg3^−/−^ mice, lacking self-tolerance against naturally expressed Dsg3 (Koch et al., 1997), were immunized against Dsg-3 to generate a source of Dsg3-reactive T and B cells, which were then isolated and injected into immune-deficient (Rag2^−/−^) but Dsg3^+/+^ recipients to induce a Dsg3-specific autoimmune response *in vivo* (Amagai et al., 2000; Tsunoda et al., 2003; Aoki-Ota et al., 2004). The recipient mice developed oral erosions with suprabasal acantholysis that was induced by the stable production of a panel of Dsg3-specific autoantibodies (Ohyama et al., 2002). A deeper analysis of the active pemphigus mouse model revealed that, in these mice, a single Dsg3-specific CD4^+^ T-cell clone was able to induce a clinical phenotype in recipient mice by activating Dsg3-reactive B cells (Takahashi et al., 2008).

A major criticism of the passive-transfer and active pemphigus mouse models is that they cannot take account of the genetic components of pemphigus predisposition, which are known from human studies to be significant determinants of disease. In response, a humanized HLA-class II transgenic mouse model of PV was developed (Eming et al., 2014). In this mouse, antigen presentation to CD4^+^ T cells is restricted to human HLA alleles, which allows the characterization of the loss of self-tolerance against human Dsg3 in an HLA-restricted *in vivo* model system (Eming et al., 2014). The immunization of HLA-DRB1*04:02-transgenic mice with immunodominant Dsg3 peptides generates a CD4^+^ T cell-dependent immune response against human Dsg3 along with the production of pathogenic Dsg3 reactive IgG antibodies (Eming et al., 2014). This model therefore allows investigation of the communication between B cells and T cells that underpins the production of anti-Dsg3 IgG, leading to the discovery that the use of anti-CD40L or the depletion of CD4^+^ T cells abolishes the induction of pathogenic anti-Dsg3 IgG. Furthermore, when Treg were induced in these mice by injection with anti-CD28 antibodies, researchers observed a reduced humoral Dsg3-specific immune response, which supports the hypothesis that the Dsg3-specific CD4^+^ T-cell dependent immune pathogenesis of PV is modulated by Treg (Schmidt et al., 2016).

Whilst these studies, in conjunction with clinical data, have supported major steps forward in our understanding and treatment of pemphigus in recent years, significant challenges remain. For example, our knowledge of the initiation of pemphigus is severely limited as we lack a spontaneous model of the disease. Similarly, our understanding of immune-pathogenesis is almost completely restricted to Dsg3 as the target autoantigen, despite knowledge from human patients that this is only one part of the pemphigus picture. A pressing need in the field is the development of a model that reproduces the full complexity of the disease, including multi-pathogenic mechanisms that target different autoantigens. The impact of the lack of such a model is nowhere clearer than in the development of novel therapeutics to treat pemphigus disease.

## Pemphigus Treatment

Prior to the era of systemic corticosteroids, 75% of patients with pemphigus were dying within the first year of the disease (Bystryn, 1996). Complications such as infections or nutritional deficiency contributed significantly towards this high mortality. The introduction of systemic corticosteroids and subsequent use of other immunomodulatory or immunosuppressive agents has brought a marked improvement in survival (Bystryn, 1996), however the level of immunosuppression required to ameliorate symptoms has created its own problems. More recently, advances in our understanding of the pathogenesis of pemphigus and the biological mechanisms of therapeutics have led to a paradigm shift in the treatment of this disease, from blanket immunosuppression towards a more targeted restriction of autoimmunity.

### Conventional Systemic Immunosuppressants/Immunomodulators

Immunosuppression with systemic corticosteroids in the form of oral prednisone or prednisolone frequently remain either the initial or mainstay of treatment for pemphigus. Different dosing schedules are employed depending on the severity of the disease: they are typically initiated at a dose of 0.5 to 1.5 mg/kg/day to achieve initial control of the disease (Hertl et al., 2015; Harman et al., 2017); while more extreme cases may be treated with either intravenous pulse methylprednisolone (500–1,000 mg daily for three to five consecutive days) or dexamethasone 100 mg daily for three consecutive days, with or without concomitant cyclophosphamide (Pasricha et al., 1995; Werth, 1996; Saha et al., 2009). Corticosteroids are effective in autoimmune diseases as they exert strong anti-inflammatory effects and induce apoptosis of lymphocytes (Shimba and Ikuta, 2020). However, as morbidity and mortality from cumulative long-term corticosteroid use is significant (Razzaque Ahmed and Moy, 1982), various non-steroid immunosuppressive agents have been trialled in a bid to improve outcomes in these patients.

Initial attempts to treat pemphigus with non-steroid immunosuppressive agents such as azathioprine, mycophenolate mofetil and cyclophosphamide achieved mixed results: when analysed together, they seemed to reduce the risk of relapse in pemphigus (Atzmony et al., 2015). However, these medications individually were not shown to be better in achieving remission, reducing deaths or reducing relapse compared to corticosteroids alone (Martin et al., 2009). Nonetheless, both azathioprine and cyclophosphamide showed steroid-sparing effects and mycophenolate demonstrated a significant effect on disease control (Martin et al., 2009). Azathioprine is often used at a dose of 2–3 mg/kg/day, mycophenolate mofetil at a dose of 2–3 g/day and cyclophosphamide at 75–150 mg/day orally or 500–1,000 mg monthly intravenously. Due to its toxicity, cyclophosphamide is not widely used and is usually only considered in severe recalcitrant cases where other therapeutic options are not available or are contraindicated (Atzmony et al., 2015).

Methotrexate, a dihydrofolate reductase inhibitor, is the earliest steroid-sparing agent used in the treatment of pemphigus. It fell out of favour due to its associated toxicities when used at higher dose. It was not until later, when low to moderate doses (ranging 15–20 mg/week) were employed that it re-emerged as a safe and effective adjunct therapy in pemphigus (Gürcan and Razzaque Ahmed, 2009). Dapsone, with its ability to interfere with neutrophil chemo-attractants, is often used in the treatment of pemphigus with a predominantly neutrophilic infiltrate, such as pemphigus foliaceus, IgA pemphigus and pemphigus herpetiformis (Kasperkiewicz et al., 2014; Porro et al., 2019; Kridin et al., 2020). Dapsone is also an appropriate adjunctive steroid-sparing agent for the treatment of patients with PV whose disease was initially controlled with corticosteroids (Werth et al., 2008).

#### Anti-CD20 Antibody Therapy

The latest game changer in the treatment of pemphigus is rituximab, a murine-human chimeric anti-CD20 monoclonal antibody (human Fc portion associated with a murine variable region) which targets B cells expressing CD20, and was initially developed and used to treat B cell malignancies (Grillo-López, 2000). First trialled in PNP in 2001 (Heizmann et al., 2001) and in PV in 2002 (Salopek et al., 2002), rituximab is now considered by many to be the primary therapeutic option in pemphigus. Injection of rituximab induces rapid depletion of B cells, including autoreactive B cells, from the peripheral blood of patients, with the B cell pool being reconstituted over the next 6–12 months from non-depleted progenitors (Mouquet et al., 2008). CD20 is expressed on the surface of B cells from the late pre-B-cell stage in the bone marrow, through naïve follicular B cells and into the memory B cell population; but the long-lived plasma cells do not express this molecule and survive in specific niches including the bone marrow, gut-associated lymphoid tissue (GALT) and skin-associated lymphoid tissue (iSALT) (Lightman et al., 2019; Zhou et al., 2020). Thus, depletion of mature B cells and short-lived plasma cells in patients with PV leads to lowering of autoreactive anti-Dsg antibodies in the serum, while antibodies produced by long-lived plasma cells, such as those directed against tetanus and pneumococcus, remain unchanged (Mouquet et al., 2008). Although rituximab is more expensive than broad spectrum immunosuppressants, a recent study showed that the initially higher cost of rituximab was almost completely off-set by costs related to management of flares and relapses in patients treated with the standard corticosteroid regimen (Hébert et al., 2020).

The therapeutic efficacy of rituximab treatment in patients with pemphigus seems to operate on multiple levels. Prolonged and continuous repopulation of naïve B cells bearing a new repertoire and a markedly delayed reappearance of memory B cells are seen after rituximab treatment: numbers of CD19^+^ B-lymphocytes even 6 years after treatment are much lower than at baseline (Colliou et al., 2013). This prolonged blockage of B cell maturation also inhibits the IgM to IgG class switching process, thereby reducing levels of autoimmune IgG^+^ circulating B lymphocytes and autoantibodies (Colliou et al., 2013): such long-lasting modification of the naive/memory-B-cell ratio accounts for the prolonged therapeutic effect of rituximab in patients with pemphigus. In addition, transitional B-cell and IL-10-secreting regulatory B cell (B reg) populations seem to expand during the B cell repopulation (Colliou et al., 2013); this could be significant because B reg cells can down-regulate inflammation and may be involved in the maintenance of long term immune tolerance (Lund and Randall, 2010; Mauri and Menon, 2017; Cao et al., 2019).

However, post-rituximab relapses do occur, and are thought to be linked to the re-emergence of anti-Dsg B cell clones that have lost self-tolerance (Hammers et al., 2015). Relapse occurs in more than 80% of PV patients over a median period of 79 months (Colliou et al., 2013), and is more likely when patients have severe disease at treatment outset and/or persistently high anti-Dsg1/3 antibody levels 3 months after treatment (Mignard et al., 2020): conversely, older patients and those given higher doses of the drug are less likely to experience early relapse (Kushner et al., 2019). It is plausible that higher doses of rituximab achieve a deeper B cell depletion and that the weaker immune systems of the elderly make the re-emergence of anti-Dsg B cell clones less likely. Alongside, long-term follow-up of auto-reactive B cells and antibodies in rituximab-treated patients with pemphigus demonstrated a complex regulatory process: in patients in remission, there were fewer autoreactive B cells than in patients with active pemphigus, and within that B cell population there was a higher proportion of IgM Dsg3^+^ cells than IgG Dsg3^+^ cells, in association with a rearrangement in Ig repertoire which had switched from an oligoclonal to polyclonal profile (Colliou et al., 2013; Hébert et al., 2019). In addition, the remaining autoreactive anti-Dsg antibodies detected in patients in remission seem to target non-pathogenic epitopes of Dsg (Müller et al., 2010).

It may be possible to reduce relapse rates, or lengthen the time to relapse, by modifying the approach to the use of rituximab in pemphigus patients. In a recent randomised control trial, 89% of patients treated with two infusions of 1 g of rituximab given a fortnight apart at baseline, and with 0.5 g at 12 and 18 months, combined with short-term prednisolone (0.5–1.0 mg/kg/day for 3–6 months) were in complete remission off therapy at 2 years, compared with only 34% of those given prednisone alone (1.0–1.5 mg/kg/day) (Joly et al., 2017). Importantly, the cumulative prednisolone dose used, and the number of severe adverse events in the rituximab-treated group, was three times and two times lower respectively, when compared to the prednisone-only group.

Alongside the relapse-rate, unfortunately, 10–20% of patients with pemphigus seem to be resistant to rituximab therapy (Joly et al., 2017). Trying to understand this phenomenon, a recent study showed that memory and germinal autoreactive B cells may persist in lymphoid tissues or ectopic lymphoid-like structures in PV lesions in cases of rituximab resistance (Zhou et al., 2020). Furthermore, the authors showed similar persistence of autoreactive CD4^+^ Th cells, which provide a crucial help to B cells for the secretion of autoantibodies. Post rituximab treatment, the disruption of immune tolerance could lead to the appearance of new autoreactive B cells, and the non-depletion of the long-lived autoreactive plasma cells with persistent production of anti-Dsg 3 antibodies could similarly explain the resistance to treatment in some patients (Hammers et al., 2015). There may also be a link between the production of human anti-chimeric antibodies (HACA) to the murine fragments of rituximab which may hamper the effectiveness of subsequent doses and contribute to a lack of therapeutic response (Lunardon and Payne, 2012). In addition, a downregulation of CD20 in some CD27^+^ memory B cells and the presence of an alternative transcript of the CD20 (D393–CD20), which was described in lymphoma and pemphigus could explain an impairment of the rituximab binding and the resultant lack of B cell depletion (Gamonet et al., 2014). Taken together, multiple pathways of incomplete/non-responsiveness to rituximab treatment have been suggested, and ways of overcoming these limitations and/or new therapies remain urgently needed.

Rituximab’s targeting of B-cells also has the side-effect of leaving patients highly susceptible to bacterial infections, particularly those of the respiratory tract and skin (Goh et al., 2007; Kamran et al., 2013). Similarly, viral infections or reactivation of latent viruses such as herpes simplex (HSV), cytomegalovirus (CMV), hepatitis B (HBV) and hepatitis C (HCV) viruses have been described in lymphoma or pemphigus patients who received rituximab (Suzan et al., 2001; Goh et al., 2007; Yeo et al., 2009; Nooka et al., 2011). Screening or prophylaxis for HSV and CMV infections is generally not regarded as necessary, but pre-treatment screening for HBV and HCV is (Sagnelli et al., 2012; Pattullo, 2015; Reddy et al., 2015). However, establishing a direct causal link between infections and rituximab therapy is often confounded by the concomitant use of other immunosuppressive agents and generalised immune dysfunction induced by the underlying diseases for which rituximab is used as therapy. As rituximab does not affect CD4^+^/CD8^+^ T-cell circulating numbers, susceptibility to bacterial infection and opportunistic infections after rituximab therapy may then be partly explained by the disruption of B cell’s role in T-cell activation and optimal CD4^+^ memory response. Hypogammaglobulinemia, which has been associated with multiple infusions of rituximab for rheumatic autoimmune diseases and lymphoma, is a risk factor for serious infections (Casulo et al., 2013; Yusof et al., 2019). However, hypogammaglobulinaemia has not been reported in pemphigus patients and it remains to be seen with longer term follow up of pemphigus patients undergoing multiple rituximab infusions. Concerns of progressive multifocal leukoencephalopathy has largely been reassured by the findings of post-marketing surveillance that this rare opportunistic infection is mainly seen in oncology (lymphoma) patients (Focosi et al., 2019).

The use of rituximab has undeniably revolutionised the treatment of pemphigus for many patients but its high initial cost, the high rate of relapse (Colliou et al., 2013) lowered resistance to infection and further lower immunogenicity to several vaccines remain significant drawbacks. These factors have driven more research aiming to discover and develop more targeted therapeutics with improved safety and efficacy profiles.

#### Adjunct Therapies

In the absence of a single therapy that works across all patients with pemphigus, different treatments have been trialled in combination with more conventional therapies, aiming to enhance the overall effect. One such adjunct therapy for severe recalcitrant pemphigus is high dose intravenous immunoglobulins (IVIG), given at 2 g/kg across five daily doses per month (Amagai et al., 2009) in addition to conventional immunosuppressive agents or rituximab (see below) (Jolles, 2001; Grando, 2019). Initial IVIG therapy rapidly elevates total circulating Ig levels, which is thought to stimulate homeostatic antibody-catabolic mechanisms, leading to lowering of serum levels of IgG1 and IgG4 anti-Dsg 1 and Dsg 3 antibodies (Green and Bystryn, 2008) while normal antibodies are replaced by those in subsequent IVIG doses: this is a particularly useful treatment when fast onset of therapy and lower risk of infection is needed. Combining rituximab and IVIG can be effective for the treatment of refractory pemphigus cases and may even induce long-term complete remission with lower risk of infection (Hamadah et al., 2019): accordingly, a clinical trial was started in June 2020 to evaluate the efficacy and safety of early use of rituximab with or without IVIGs in patients with moderate to severe pemphigus (NCT04400994). Immunoadsorption, which also rapidly removes anti-Dsg IgG from the circulation, has also been trialled in conjunction with conventional immunosuppressive treatment and intravenous rituximab (Eming and Hertl, 2006; Behzad et al., 2012). In its current form, immunoglobulins are non-specifically adsorbed and removed, but studies are underway to develop anti-Dsg immunoglobulin-specific adsorption (Langenhan et al., 2014); it is, however, not easily available in many parts of the world, limiting its potential for widespread use.

Complementary to systemic therapeutic options, local treatment of mucosal - especially oral - lesions of pemphigus should not be overlooked. Poor oral hygiene can be a contributory factor for persistent oral erosions in pemphigus (Gambino et al., 2014), and topical analgesics or anaesthetics (e.g., lidocaine 2% gel) can be used prior to eating or brushing of teeth. Topical medium or high potency corticosteroids (e.g., clobetasol propionate 0.05% in adhesive paste) can also be applied directly to the lesions (Lozada-Nur et al., 1994) or used as mouth gargle. Alongside, the use of topical calcineurin inhibitors and cyclosporine mouthwash may be also beneficial (Gooptu and Staughton, 1998; Hodgson et al., 2003). Candida infection is a common occurrence in patients with mucosal pemphigus who are treated with topical or systemic glucocorticoids (Lozada-Nur et al., 1994): oral nystatin swish-and-swallow or mouthwash can be used as prophylaxis. Dietary advice in favour of a soft diet and the avoidance of spicy or very hot foods can also be helpful for patients.

## Novel Treatment in the Pipeline

As we learn more about the pathogenesis of pemphigus, the development of more targeted therapeutic approaches with improved safety and efficacy profiles is gradually becoming possible.

Recent studies characterising the immune features of pemphigus lesions themselves have paved the way for innovative local treatment strategies. For example, intralesional delivery of rituximab, which has already been used in the treatment of lymphoma (Davies et al., 2017), has been proposed as a way of targeting the diffuse ectopic lymphoid-like structures that are commonly seen in lesions of both PV and PF and to treat refractory oral pemphigus vulgaris (Vinay et al., 2015; Zhou et al., 2020). Disruption of these lymphoid-like structures, which are composed of T cells, dendritic cells, centroblasts, plasmablasts and plasma cells might therefore disrupt the niche that supports the *in situ* B cell differentiation, clonal expansion and production of autoreactive antibody in the skin of patients with pemphigus. Identification of the cell composition and exploration of the impact of the injection of steroids into lesions of patients with pemphigus that harbour ectopic lymph node-like structures is ongoing (NCT04509570).

Some novel approaches target specific obstacles within current therapeutic settings. Aiming to restore the efficacy of anti-CD20 therapy in rituximab patients with HACA, subcutaneous injection of Ofatumumab, a fully human anti-CD20 IgG_1_ antibody with increased binding affinity for CD20, has been successfully trialled in a single patient (Rapp et al., 2018), but has yet to be assessed in a larger cohort. Similarly, veltuzumab, a second generation humanized anti-CD20 antibody, has been successfully used in a case of rituximab-refractory PV (Ellebrecht et al., 2014). The development of these second and third generation anti-CD20 antibodies, which possess superior B cell-depleting qualities and higher binding affinities compared to rituximab, might represent an important step forward in the treatment of pemphigus and other autoimmune B cell diseases.

A different type of treatment obstacle can be simply a practical limitation, as in the case of IVIG: these products are generally considered safe and effective, but their production requires an abundant supply of human plasma in order to generate the large doses of product (up to 2 g/kg body weight) needed for therapy.

To overcome this supply limitation, antibodies with high affinity for the neonatal Fc receptor (FcRn) have been developed. These antibodies represent a key determinant actor for IgG levels and functions (Pyzik et al., 2019). FcRn-targeting therapeutics aim to block the binding of IgG and IgG immune complexes to the FcRn, thereby accelerating their breakdown and inducing a reduction in overall plasma IgG levels, including the levels of pathogenic autoantibodies (Blumberg et al., 2019).

Data from a phase 1b/2a study using ALXN1830 (NCT03075904), a humanized affinity-matured IgG4-kappa monoclonal antibody with high affinity for the neonatal Fc receptor, has shown major improvements in both cutaneous and mucosal disease, and an overall acceptable safety and tolerability profile (Werth et al., 2021). In this trial, ALXN1830-associated clinical improvement was accompanied by a similarly rapid and significant decrease in levels of total IgG, all individual IgG subclasses, and IgG immune complexes. In addition, this study thereby provides evidence that IgG circulating immune complexes may be involved in the pathogenesis of pemphigus. Another promising anti-FcRn therapeutic is also being trialled in pemphigus. Efgartigimod is a human IgG1 antibody Fc-fragment, a natural ligand of FcRn, that has been engineered for increased affinity to FcRn compared with endogenous IgG (Zuercher et al., 2019). Proven safe and effective in patients with myasthenia gravis (Howard et al., 2021), results from a completed phase two trial of efgartigimod in patients with PV (NCT03334058) have shown that it is well-tolerated and exhibited an early effect on disease activity and outcome parameters, providing support for further evaluation as a therapy for pemphigus (Goebeler et al., 2021). Two phase 3 clinical trials assessing the early and long-term efficacy and safety of a subcutaneous formulation of efgartigimod in adults with pemphigus is on-going (NCT04598451 and NCT04598477).

Focussing on the immune-associated SNPs that have been linked with pemphigus, Assaf S et al. recently demonstrated through a series of experiments on how ST18 contributed to destabilization of cell-cell adhesion in a tumour necrosis factor (TNF)-α-dependent manner, potentially opening up new therapeutic option of using TNF-α inhibitors in the treatment of pemphigus (Assaf et al., 2021).

Targeting another aspect of the biology of B cells, such as their survival or their differentiation into plasma cells, is a useful approach that has been tested in SLE and could be applied to the treatment of pemphigus. The use of belimumab, a monoclonal human IgG1 antibody that binds to soluble B lymphocyte stimulator (BLyS) or B cell activating factor belonging to the TNF family (BAFF) (Möckel et al., 2021), or atacicept, a fully human recombinant fusion protein that blocks BLyS and the proliferation-inducing ligand (APRIL) (Kaegi et al., 2020), might be a promising treatment of pemphigus. This is further supported by the recent finding that a modification of the BAFF/BAFF receptor axis in patients with pemphigus could explain the high number of relapses following standard corticosteroid treatment alone versus with rituximab (Hébert et al., 2021). New therapies such as Bruton’s tyrosine kinase (BTK) inhibitors have also emerged as a potential treatment option: BTK inhibitors seem to be able to neutralize pathogenic autoantibodies, to inhibit new autoantibody production and to possess anti-inflammatory effects (Weber et al., 2017). A trial involving the use of a BTK inhibitor, PRN 1008, in patients with PV (NCT02704429) has been completed and recently published (Murrell et al., 2021). The study suggests that BTK inhibition may be a promising treatment strategy and supports further investigation of such inhibitor for the treatment of pemphigus.

Recently, chimeric antigen receptor (CAR) T cell technology has revolutionized cancer immunotherapy (reviewed in Mohanty et al. (2019)). This approach uses T cells from the patient’s own blood that are genetically manipulated in research laboratories to express a CAR capable of recognizing a specific cell type uniquely expressing the target antigen. In the case of pemphigus, researchers have engineered a chimeric autoantibody receptor (CAAR), with Dsg3 as the extracellular domain in order to generate CAAR-T cells that recognize the Dsg3-specific BCR on autoreactive B cells and induce their elimination (Ellebrecht et al., 2016). Preclinical study has demonstrated that DSG3-CAART could be a precise therapy for PV (Lee et al., 2020). A phase one clinical trial to determine the maximum tolerated dose of Dsg3-CAART in mucosal dominant PV patients is ongoing (NCT04422912). Whilst this is potentially a promising step forwards, the efficacy of Dsg3-CAAR-T cell therapies in pemphigus may be limited by the single antigen focus, and so the repertoire of recognition might need to be expanded in future trials if this approach is found to be safe and well-tolerated.

## Vaccination and Pemphigus

Vaccination strategy and Pemphigus treatment are in need of more studies especially in this time of COVID-19 pandemic. The effect of rituximab and other anti-CD20 monoclonal antibodies on vaccine response has been studied for inactivated vaccines (Baker et al., 2020). These studies have suggested that rituximab recipients mount attenuated yet meaningful vaccine responses. Concerning live attenuated vaccines, no study addressing their immunogenicity has been started due to the safety concerns regarding the use of these vaccines in rituximab recipients. In addition, no studies have evaluated yet the safety and immunogenicity of messenger RNA vaccines or viral vector vaccines, which are among the leading COVID-19 vaccine candidates. To gain knowledge on COVID-19 vaccine and pemphigus, a clinical trial has been started recently in august 2021 to compare the immune response to different COVID-19 vaccine booster doses (Moderna COVID-19 vaccine, Pfizer-BioNTech COVID-19 vaccine, or Janssen COVID-19 vaccine) in participants with autoimmune disease requiring immunosuppressive medications, including pemphigus patients (NCT05000216).

## Conclusion

Alongside advances in our understanding of pemphigus pathogenicity, the number of therapeutic options to treat pemphigus has increased over the last decade, with more targeted therapies and refined diagnostic techniques beginning to emerge. However, with better definition of the clinical subtypes of pemphigus, the aetiology and immune pathogenesis of these diseases is revealed to be more and more complex, and still requires further investigation. Better comprehension of the early stages of pemphigus, the role of innate and adaptive immune cells - most notably dendritic cells and full analysis of the involved B cells’ biology will be required. To support this, the field should develop new mouse models that incorporate all the immune players necessary for the emergence of pemphigus variants, and that can be used to test new and innovative therapies.
